# Administration of a recombinant secretory leukocyte protease inhibitor prevents aortic aneurysm growth in mice

**DOI:** 10.1007/s11010-025-05374-0

**Published:** 2025-08-29

**Authors:** Aika Yamawaki-Ogata, Masato Mutsuga, Yuji Narita

**Affiliations:** Department of Cardiac Surgery, Nagoya University Graduate School of Medicine, 65 Tsurumai-Cho, Showa-Ku, Nagoya, Aichi 466-8550 Japan

**Keywords:** Aortic aneurysm, Mesenchymal stem cells, Secretome, Secretory leukocyte protease inhibitor, Inflammation

## Abstract

Pharmacological interventions to inhibit the progression of aortic aneurysm (AA) have not yet been established. We previously reported that mesenchymal stem cells (MSCs) provide a potential foundation for less invasive treatment of AA. In this study, we investigated the secretory proteins from MSC supernatants to clarify the therapeutic effects of MSCs. Furthermore, we treated thoracoabdominal aortic aneurysm (TAAA) mice with two anti-inflammatory proteins from among these secretory proteins to confirm their therapeutic effects. Protein profiles of MSC-secreted factors were analyzed using protein microarrays, and two anti-inflammatory proteins, namely progranulin (PGRN) and secretory leukocyte protease inhibitor (SLPI), were identified. Apolipoprotein E-deficient mice were continuously infused with angiotensin II via an osmotic pump for 4 weeks to induce TAAA formation, and then recombinant rPGRN and/or rSLPI were administered intraperitoneally. Mice were sacrificed at 8 weeks, and aortas were analyzed for protein expression and also stained with Elastica van Gieson and immunofluorescence to detect inflammatory cells. Intraperitoneal administration of rSLPI inhibited TAAA growth more than rPGRN alone or the combination of rPGRN and rSLPI, by inducing the following effects: downregulation of inflammatory cytokines and chemokines, specifically IL-1β, IL-6, TNF-α, and MCP-1; reduced NO production; decreased phosphorylated NF-κB levels; and decreased elastin destruction and infiltration of inflammatory cells. We identified anti-inflammatory proteins, including PGRN and SLPI, in the MSC supernatants and showed that the administration of rSLPI inhibited TAAA progression in mice. These promising preliminary data present a new approach for the treatment of less invasive TAAA.

**Graphical abstract:**

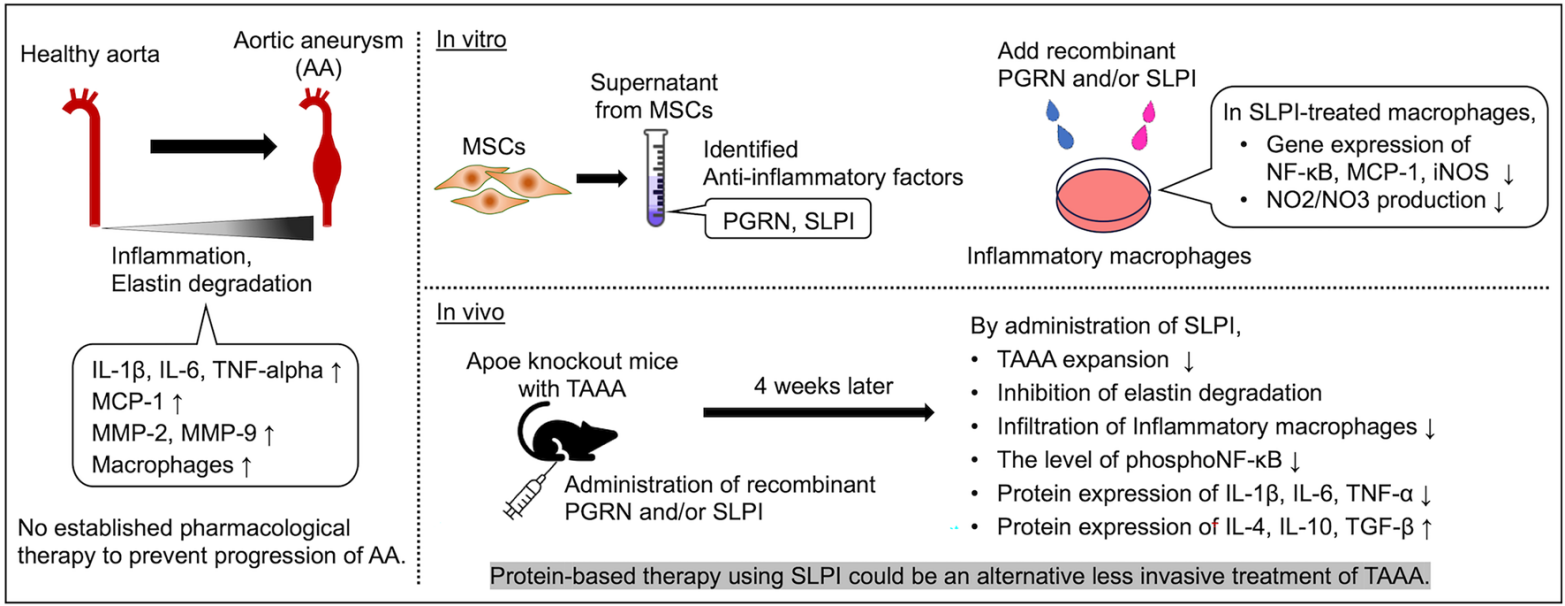

**Supplementary Information:**

The online version contains supplementary material available at 10.1007/s11010-025-05374-0.

## Introduction

Aortic aneurysm (AA) that dilates beyond 1.5 times the width of a normal aorta may undergo further dilation with few symptoms. While prophylactic surgical repair effectively prevents rupture, in high-risk individuals such as older, frail patients, the risks of surgical morbidity and mortality are increased [1]. Although recent endovascular aortic repair techniques effectively prevent rupture and are less invasive than previous approaches, they still have several limitations related to anatomy, migration, and endoleaks [2, 3]. There is no established clinical pharmacological therapy to prevent AA dilation. Thus, it is necessary to develop a new, less-invasive strategy to protect against AA growth and rupture.

Expanding AA is characterized by a number of proinflammatory conditions, including the following: infiltration of inflammatory cells; upregulation of inflammatory cytokines and chemokines, including interleukin (IL)−1β, IL-6, and tumor necrosis factor-α (TNF-α); increased matrix metalloproteinase (MMP) activity; and elastin degradation [4]. Currently, mesenchymal stem cell (MSC)-based therapy has been proven to be safe and effective in clinical trials in a number of fields, such as cardiology and immunology [5, 6]. Regarding AA treatment, we previously reported that AA shrunk transiently due to the anti-inflammatory properties of intravenously administered MSCs and discussed the possibility of a paracrine mechanism [7, 8]. Supernatant from MSCs is considered to be an abundant source of secretory factors and currently serve as a cell-free therapy for various diseases. However, the MSC-derived secretome consists of diverse components with different biological effects, and it remains unclear which bioactive factors are effective against specific diseases.

In this study, we investigated protein profiles of the MSC-derived secretome to identify bioactive factors (particularly anti-inflammatory factors) that may be effective for the treatment of AA. In addition, we administered two recombinant proteins, namely progranulin (PGRN) and secretory leukocyte protease inhibitor (SLPI), to thoracoabdominal aortic aneurysms (TAAA) mice and determined their therapeutic effects against TAAA.

## Methods

### MSC culture and protein microarray analysis

Mouse bone marrow-derived MSCs were obtained using the methods described in our previous studies [9]. MSCs at passage 6 were cultured with Dulbecco’s modified Eagle’s medium (DMEM) supplemented with 10% fetal bovine serum (FBS) and 1% antibiotic (100 U/mL penicillin G, 100 µg/mL streptomycin, and 0.25 µg/mL amphotericin B; Gibco), and incubated at 37 °C in a 5% CO_2_ atmosphere. After cells were 80% confluent, they were washed with phosphate-buffered saline (PBS) and replaced with FBS-free DMEM, then starved for 48 h. MSC supernatant was collected and cleared by centrifugation for 5 min at 1500×*g*. The supernatant was concentrated 20-fold using an Amicon Ultra-4 filter with a 3-kDa molecular weight cut-off (Merck Millipore, Darmstadt, Germany). Supernatant without MSCs was also collected as a negative control using the same procedure. The concentration of total protein was measured using a Qubit protein assay kit on a Qubit 2.0 fluorometer (Invitrogen, Carlsbad, CA, USA). Total protein (0.4 mg) was added to a well of an array slide. The protein microarrays, which can identify 308 different mouse proteins by direct biotin labeling, were processed using L-308 Mouse Antibody Arrays (RayBiotech Life, Norcross, GA, USA).

### Animals and TAAA models

Experimental procedures followed the institutional guidelines for the care and use of laboratory animals and the ARRIVE guidelines. All experiments and procedures were approved by the Animal Experiment Advisory Committee of the Nagoya University School of Medicine (Protocol No. 20103). For this study, male apolipoprotein E deficient (apoE^−/−^) mice were purchased from the Jackson Laboratory (Sacramento, CA, USA).

TAAA was induced in forty 28-week-old male apoE^−/−^ mice by infusion with angiotensin II (ATII; 1000 ng/kg/min, Calbiochem, Darmstadt, Germany) for 4 weeks through subcutaneous osmotic mini-pumps (model 2004; DURECT, Cupertino, CA, USA) [7]. All mice underwent infusion and maintenance anesthesia with 1.5% isoflurane (FUJIFILM Wako Pure Chemical, Osaka, Japan).

### Intraperitoneal injection of rPGRN and/or SLPI

Confirmation of TAAA formation in mice was performed by echography using a LOGIQ e Premium ultrasound scanner and a 10–22 MHz probe (GE Healthcare, Chicago, IL, USA) at 0 and 4 weeks after ATII infusion [10]. A TAAA was defined as a dilated aorta with a diameter at least 1.5 times larger at 4 weeks than at 0 weeks following previously published guidelines [11]. Mice with TAAA were divided randomly into four groups, with each of the following injected intraperitoneally: (1) 10 mg/kg rPGRN in 0.2 mL PBS (R&D Systems, Minneapolis, MN, USA; recombinant mouse, P group, *n* = 10), (2) 10 mg/kg rSLPI in 0.2 mL PBS (R&D Systems; recombinant human, S group, *n* = 10), (3) 10 mg/kg rPGRN and rSLPI in 0.2 mL PBS (PS group, *n* = 10), and (4) 0.2 mL saline (Saline group, *n* = 10). All mice underwent echography at 6 and 8 weeks. At 8 weeks, following euthanasia with an overdose of isoflurane, the aorta was carefully exposed and photographed using a DP70 digital camera (Olympus, Tokyo, Japan).

### EVG staining

EVG staining was performed as previously described [10]. The frozen cross-Sects. (10 µm) were stained for elastic lamellae using Weigert’s resorcin-fuchsin (Muto Pure Chemicals, Tokyo, Japan). Sections were photographed with a DP80 digital camera (Olympus). Images were analyzed using Cellsens software (Olympus) to determine the area of elastin staining as the percentage area of elastic lamellae and the percentage area of the medial component between the elastic lamellae (elastin gap area), both compared to the total medial tissue area [12].

### Immunofluorescence staining

Immunofluorescence staining was performed as previously described [10]. Briefly, the primary antibodies used were rat anti-inducible nitric oxide synthase (iNOS) antibody (1:50, Santa Cruz Biotechnology, Dallas, TX, USA) and rabbit anti-CD206 antibody (1:1000, Abcam, Cambridge, MA, USA). The secondary antibodies used were anti-rat IgG Alexa Fluor 488-conjugated antibody (1:5000, Cell Signaling Technology, Danvers, MA, USA) and anti-rabbit IgG Alexa Fluor 555-conjugated antibody (1:5000, Cell Signaling Technology). Negative control experiments used rat IgG1 and rabbit IgG isotype control antibodies (Cell Signaling Technology) at the same concentrations as the primary antibodies.

### Enzyme-linked immunosorbent assay (ELISA) and measurement of endogenously active MMP-2 and MMP-9

TAAA tissues were homogenized using RIPA buffer (Fujifilm Wako Pure Chemical Corporation, Osaka, Japan). Lysate protein concentration was measured using a Qubit Protein Assay Kit and Qubit 2.0 fluorometer (Thermo Fisher Scientific). An equal concentration of total protein was applied to each enzyme-linked immunosorbent assay (ELISA) kit (IL-4, IL-10, IL-1β, IL-6, transforming growth factor (TGF)-β1, TNF-α, tissue inhibitor of metalloproteinase (TIMP)−1, and monocyte chemotactic protein (MCP)−1 (Invitrogen); and TIMP-2, c-Jun N-terminal kinase (JNK) 1/2 (total or phosphorylated JNK; tJNK or pJNK, pT183/Y185), nuclear factor-kappa B (NF-κB, total or phosphorylated NF-κB; tNF-κB or pNF-κB, p65), and phosphorylated Smad3 (pSmad3, pS423/S425) (Abcam)), and the protein amounts were determined. To measure the endogenous activities of MMP-2 and MMP-9 in aortic tissues, an equal concentration of total protein was applied to each MMP enzymatic activity assay kit (SensoLyte 520 MMP-2 assay kit: ANASPEC, Fremont, CA, USA; Mouse MMP-9 activity assay kit: QuickZyme Bioscience, Leiden, The Netherlands), and the expression levels of MMP activities were measured.

### Pretreatment of cultured macrophages with rSLPI

Cell culture and expansion of murine macrophages were performed as previously described [10]. Macrophages were plated at 2 × 10^4^ cells per well in a 96-well plate, and pretreated with rSLPI (R&D Systems) at doses of 0, 0.1, 1, or 10 μg/mL with incubation at 37 °C in a humidified atmosphere of 5% CO_2_ in air for 24 h. The medium was then replaced for 24 h with growth medium containing 10 ng/mL liposaccharide (LPS, Sigma-Aldrich, St. Louis, MO, USA) and 2 ng/mL TNF-α (recombinant human, Peprotech, Cranbury, NJ, USA). Growth medium containing LPS but not TNF-α was used as negative control. After incubation, nitric oxide (NO) production in supernatant was measured, and cells underwent RNA extraction.

### Measurement of NO production

To determine the volume of NO produced, supernatant was harvested from macrophages treated with rSLPI and measured using a NO2/NO3 assay kit-C II Colorimetric (Dojindo, Kumamoto, Japan).

### Quantitative real-time polymerase chain reaction (qRT-PCR)

Quantitative RT-PCR was performed as previously described [10]. Briefly, cDNA was synthesized using the Takara PrimeScript RT reagent kit (Takara Bio Inc., Shiga, Japan). qRT-PCR analysis was performed to determine the gene expression of IL-1β, IL-6, IL-10, iNOS, MCP-1, NF-κB, TNF-α, with β-actin (Sigma-Aldrich) as a control (Supplementary Table 1). All data were analyzed by CFX Maestro ver.1.1 Software (Bio-Rad, Hercules, CA, USA).

### Statistical analysis

Statistical significance between groups was calculated by two-way repeated measure ANOVA followed by Tukey’s multiple comparisons test, Dunnett T3 multiple comparisons test, Dunn’s multiple comparisons test, Mann–Whitney *U* test, or Wilcoxon matched-pairs signed rank test, as appropriate, using GraphPad Prism for Mac (Version 8; GraphPad Software, San Diego, CA, USA). All error bars represent standard error of the mean (SEM). Values were considered statistically different when *p* was < 0.05.

## Results

### Proteomic analysis of MSC supernatant

**Fig. 1:**
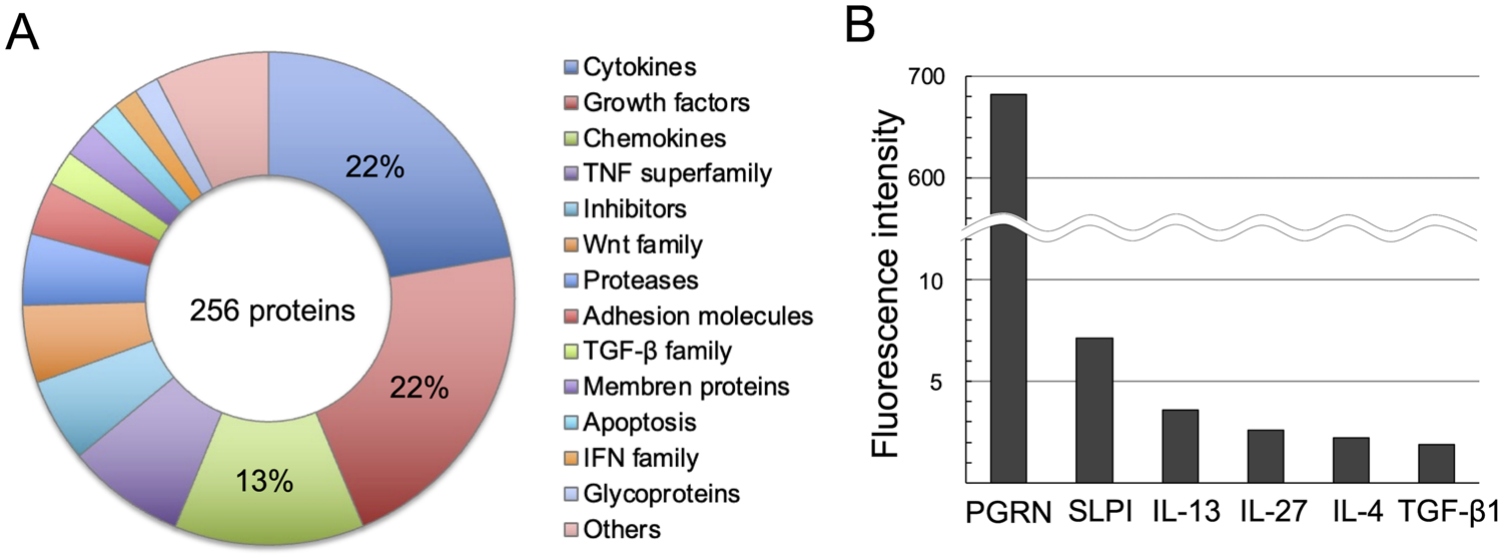
Protein microarray analysis of MSC supernatants. **A** Two hundred fifty-six proteins were identified. Cytokines, growth factors, and chemokines were also highly enriched. **B** Fluorescence intensity values of six anti-inflammatory factors: PGRN, SLPI, IL-13, IL-27, IL-4, and TGFβ−1

Commercially available protein microarrays were used to analyze the expression of secreted factors from MSC supernatant harvested under normal cell culture conditions. Comparing supernatant without MSCs as the negative control, protein microarray analysis identified 256 different proteins, including cytokines, growth factors, and chemokines, in MSC supernatant; the details are shown in Fig. 1A. Notably, the proteins included anti-inflammatory factors such as PGRN, SLPI, IL-13, IL-27, IL-4, and TGF-β1. Figure 1B shows that the fluorescence intensity of PGRN was highest among these, while that of SLPI was second highest. A map and images of the protein microarray are shown in Supplementary Fig. 1.

### Administration of rSLPI suppresses TAAA growth

**Fig. 2:**
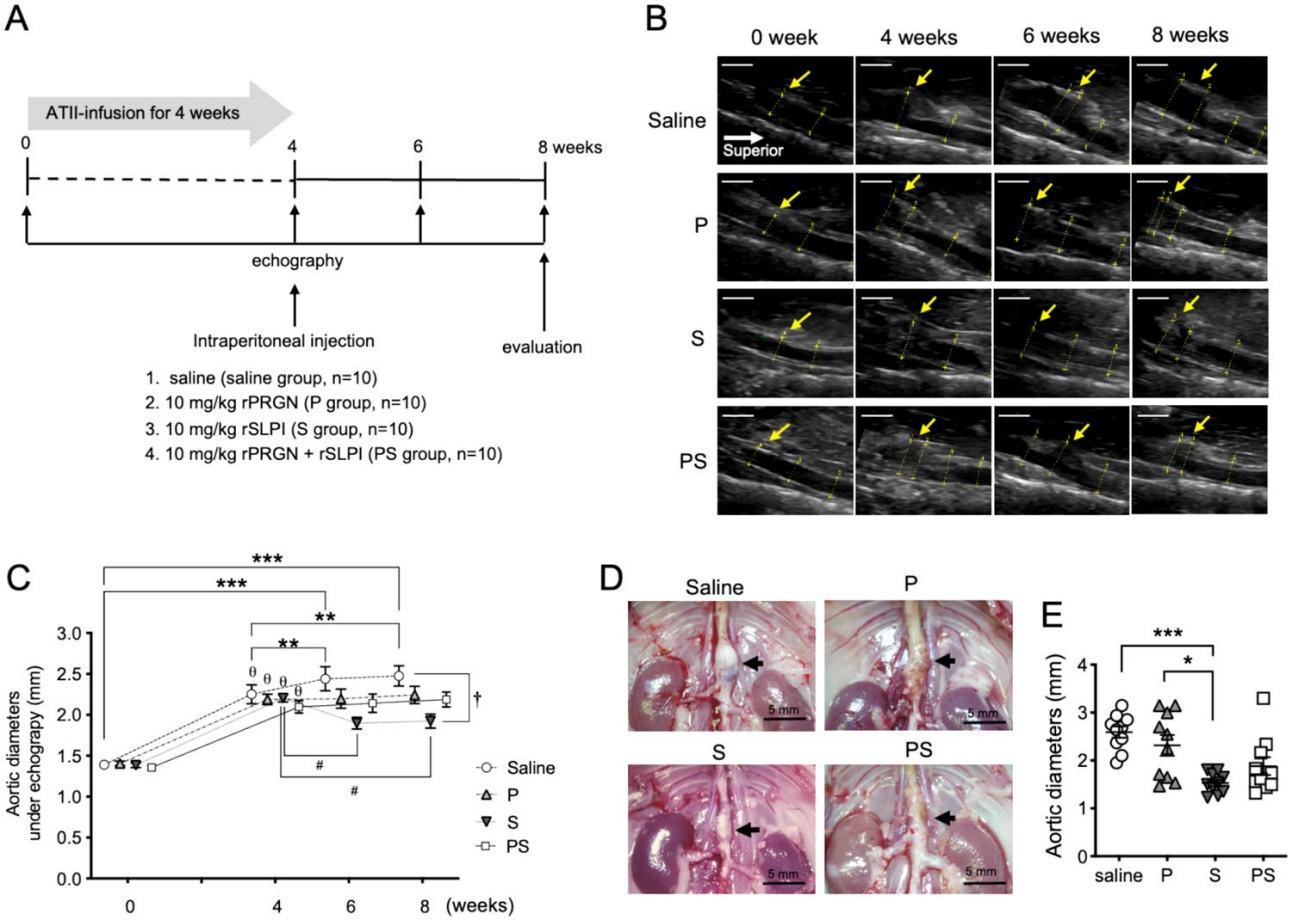
Scheme of the in vivo study protocol and aortic analysis. **A** ATII was infused into apoE^−/−^ mice for four weeks, and then 10 mg/kg PGRN, SLPI, or saline was injected intraperitoneally (n = 10 mice). The mice underwent echography at 0, 4, 6, and 8 weeks, and were sacrificed at 8 weeks. **B** Representative long-axis echographic images of the thoracoabdominal aorta. Scale bars = 2 mm. **C** Aortic diameters measured using echography (n = 10 mice per group). Data are means ± SEM. ***p < 0.001 versus just before ATII infusion (week 0) within the group. ^θ^p < 0.001 versus immediately before ATII infusion (week 0) within the group. **p < 0.05 versus ATII infusion (week 4) within the group. ^#^*p* < 0.05 versus 4 weeks in the saline group. ^†^*p* < 0.05 between groups at 8 weeks. Data were assessed using two-way repeated measures ANOVA followed by Tukey’s multiple comparison test. **D** Representative microscopic images of TAAA obtained by microscopy (black arrows). Scale bars = 5 mm. **E** Aortic diameters measured by microscopy (n = 10 mice per group). Data are means ± SEM. *p < 0.05 and ***p < 0.001 assessed by the Dunn’s multiple comparisons test. ATII: angiotensin II, PGRN: progranulin, SLPI: secretory leukocyte proteinase inhibitor

Aortic echography was performed according to the time course shown in Fig. 2A. No death, TAAA rupture, or paraplegia were observed in any of the mice in this study. The survival rate was 100% in all the groups. The infradiaphragmatic to renal portion of the thoracoabdominal aorta was visualized using echography. At 4 weeks, saccular aneurysms were identified (arrow), and their maximum diameters were measured (Fig. 2B). In all groups, aortic diameters were significantly larger (at least 1.5 times as large) at 4 weeks than at 0 weeks (Fig. 2C, *p* < 0.001). In the saline group, the mean aortic diameter was significantly larger at week 6 and 8 compared with week 4 (4 weeks: 2.25 ± 0.11 mm, 6 weeks: 2.44 ± 0.11 mm, 8 weeks: 2.48 ± 0.07 mm, 6 vs. 4 weeks: *p* < 0.05, 8 vs. 4 weeks: *p* < 0.05, Fig. 2C), whereas the P and PS groups showed no remarkable TAAA dilation. On the other hand, the mean TAAA diameter in the S group was significantly smaller at 6 and 8 weeks compared with 4 weeks (4 weeks: 2.20 ± 0.05 mm, 6 weeks: 1.90 ± 0.1 mm, 8 weeks: 1.92 ± 0.13 mm, 6 vs 4 weeks: *p* < 0.05, 8 vs. 4 weeks: *p* < 0.05, Fig. 2C). In addition, the mean aortic diameter of the S group at week 8 was significantly smaller than that of the saline group (1.92 ± 0.13 vs. 2.48 ± 0.07 mm, *p* < 0.05, Fig. 2C). Individual aortic diameter data are shown in Supplementary Fig. 2. Microscopic findings at 8 weeks revealed the formation of a TAAA involving the descending part of the thoracic aorta and abdominal aorta proximal to the renal arteries (Fig. 2D). The maximum aortic short-axis diameter was significantly smaller in the S group than in the saline and P groups (S: 1.53 ± 0.17 mm, saline: 2.59 ± 0.15, P: 2.32 ± 0.22 mm, S vs. saline: *p* < 0.001, S vs. P: *p* < 0.05, Fig. 2E).

### rSLPI ameliorates disruption of elastic lamellae and reduces infiltration of inflammatory cells

**Fig. 3:**
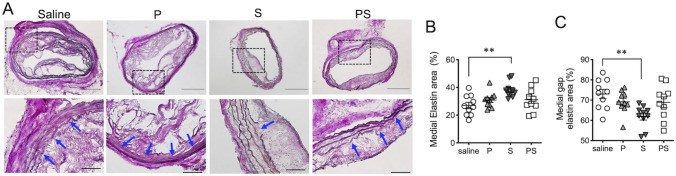
Quantitative analysis of elastin in EVG-stained samples. **A** EVG staining indicates destruction of the elastic lamellar structure (black arrows). Scale bars = 200 μm. Quantitative analysis of the medial elastic lamellae (**B**), medial gap elastic lamellae (**C**). Data are means ± SEM. **p < 0.01 assessed by the Dunn’s multiple comparisons test

EVG staining showed considerable degradation of the elastic lamellae in the saline group, with thin elastin fibers in the P and PS groups, whereas less disruption of the elastic lamellae was observed in the S group (Fig. 3A). Elastic lamellae in the media were more abundant in the S group than in the saline group (38 ± 1.7 vs. 27 ± 2.3%, respectively; *p* < 0.01, Fig. 3B). In contrast, the medial gap area was smaller in the S group than that in the saline group (62 ± 1.7 vs. 73 ± 2.3%, respectively; *p* < 0.01, Fig. 3C).

**Fig. 4:**
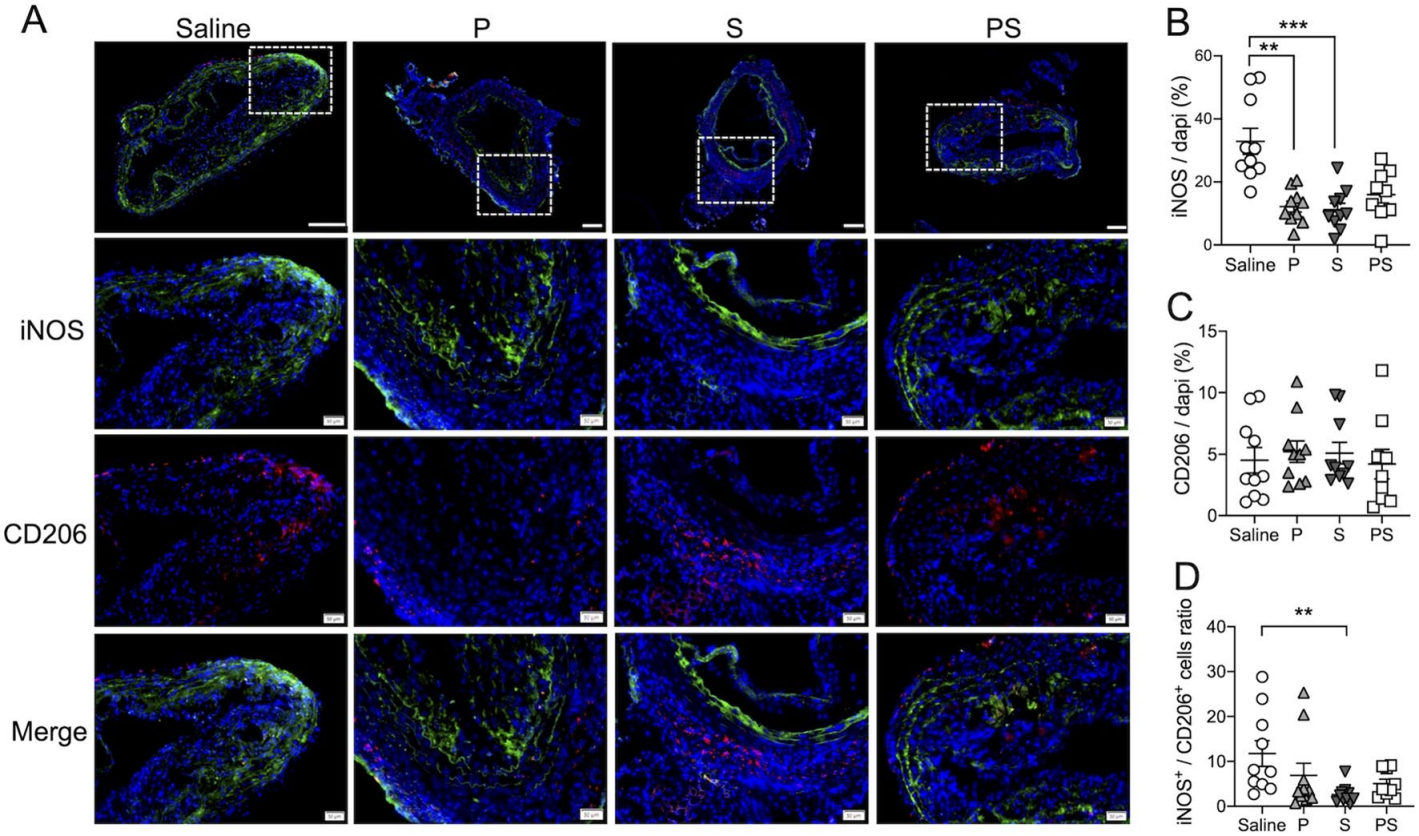
Quantitative analysis of immunofluorescence images. **A** iNOS is shown in green, whereas CD206 is shown in red. Scale bars = 100 μm. **B** Quantitative analysis of cells positive for iNOS and CD206 (**C**), and the ratio of iNOS^+^ to CD206^+^ cells (**D**). Data are means ± SEM. **p < 0.01 and ***p < 0.001 assessed by the Dunn’s multiple comparisons test

Immunofluorescence staining showed infiltration of many iNOS-positive cells, including inflammatory cells such as macrophages and neutrophils, in the atherosclerotic region and media, as well as infiltration of CD206-positive cells, including most macrophages and dendritic cells, in the adventitia (Fig. 4A). The percentage of iNOS-positive inflammatory cells was significantly lower in the S group than in the saline group (11.2 ± 2.0 vs. 30.9 ± 9.3%, *p* < 0.001, Fig. 4B), while there was no difference in the percentage of CD206-positive cells (Fig. 4C). In addition, the ratio of iNOS- to CD206-positive cells was significantly lower in the S group than in the saline group (5.2 ± 0.9 vs. 3.1 ± 0.8%, respectively; *p* < 0.01; Fig. 4D).

### rSLPI regulates inflammatory responses but not TIMPs or MMPs in TAAA tissue homogenates

**Fig. 5:**
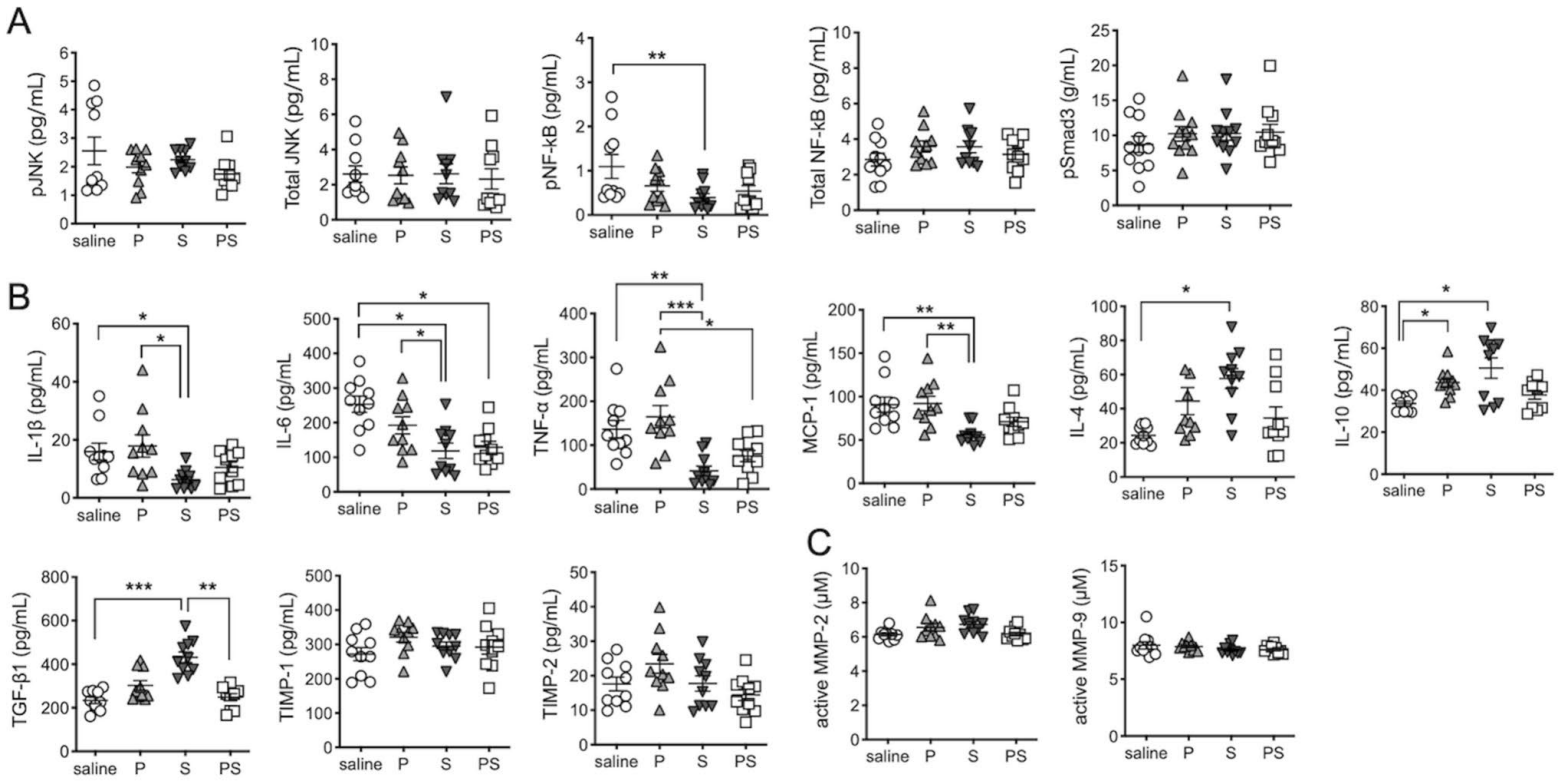
Quantitative analysis of protein expression levels in TAAA tissue homogenates. **A** Signaling pathways involving JNK, NF-kB, and smad3 (n = 10 mice each). **B** ELISA of inflammatory cytokines (IL-1β, IL-6, TNF-α), chemokines (MCP-1), anti-inflammatory cytokines (IL-4, IL-10, and TGF-β1), and TIMPs (TIMP-1 and TIMP-2) (n = 10 mice each). **C** Measurement of endogenously active MMP-2 and MMP-9 levels (n = 10 mice each). Data are means ± SEM. *p < 0.05; **p < 0.01; ***p < 0.001 assessed by the Dunn’s multiple comparisons test

An analysis of signaling pathways showed that pNF-κB was significantly downregulated in the S group compared with the saline group (0.4 ± 0.1 vs. 1.1 ± 0.3 pg/mL, *p* < 0.01, Fig. 5A), whereas there was no significant difference in the expression level of pJNK or pSmad3. Protein levels of IL-1β, IL-6, TNF-α, and MCP-1 were significantly lower in the S group than in the saline group (IL-1β, 6.3 ± 1.1 vs. 15.9 ± 2.9 pg/mL, *p* < 0.05; IL-6, 118.7 ± 22.2 vs. 253.2 ± 23.7 pg/mL, *p* < 0.05; TNF-α, 41.5 ± 11.0 vs. 136.6 ± 19.8 pg/mL, *p* < 0.01; MCP-1, 56.0 ± 3.4 vs. 90.8 ± 8.6 pg/mL, *p* < 0.01, Fig. 5B). Moreover, the expression levels of IL-1β, IL-6, TNF-α, and MCP-1 were significantly lower in the S group than in the P group (IL-1β, 6.3 ± 1.1 vs. 17.9 ± 3.8 pg/mL, *p* < 0.05; IL-6, 119 ± 22 vs. 192 ± 25 pg/mL, *p* < 0.05; TNF-α, 42 ± 11 vs. 165 ± 25 pg/mL, *p* < 0.001; MCP-1, 56 ± 3 vs. 92 ± 8 pg/mL, *p* < 0.01, Fig. 5B). In contrast, the protein levels of the anti-inflammatory factors IL-4, IL-10, and TGF-β1 were significantly higher in the S group than in the saline group (IL-4, 58 ± 6 vs. 24 ± 2 pg/mL, *p* < 0.05; IL-10, 51 ± 5 vs. 34 ± 1 pg/mL, *p* < 0.05; TGF-β1, 432 ± 25 vs. 234 ± 14 pg/mL, *p* < 0.001; Fig. 5B). There were no differences in the enzymatic activity levels of active MMP-2 or MMP-9 between the groups between groups in the enzymatic activity levels of active MMP-2 or MMP-9 (Fig. 5C), or in the protein levels of their respective inhibitors, TIMP-1 and TIMP-2 (Fig. 5B).

### rSLPI promotes downregulation of inflammatory genes and reduced NO production in cultured macrophages

**Fig. 6:**
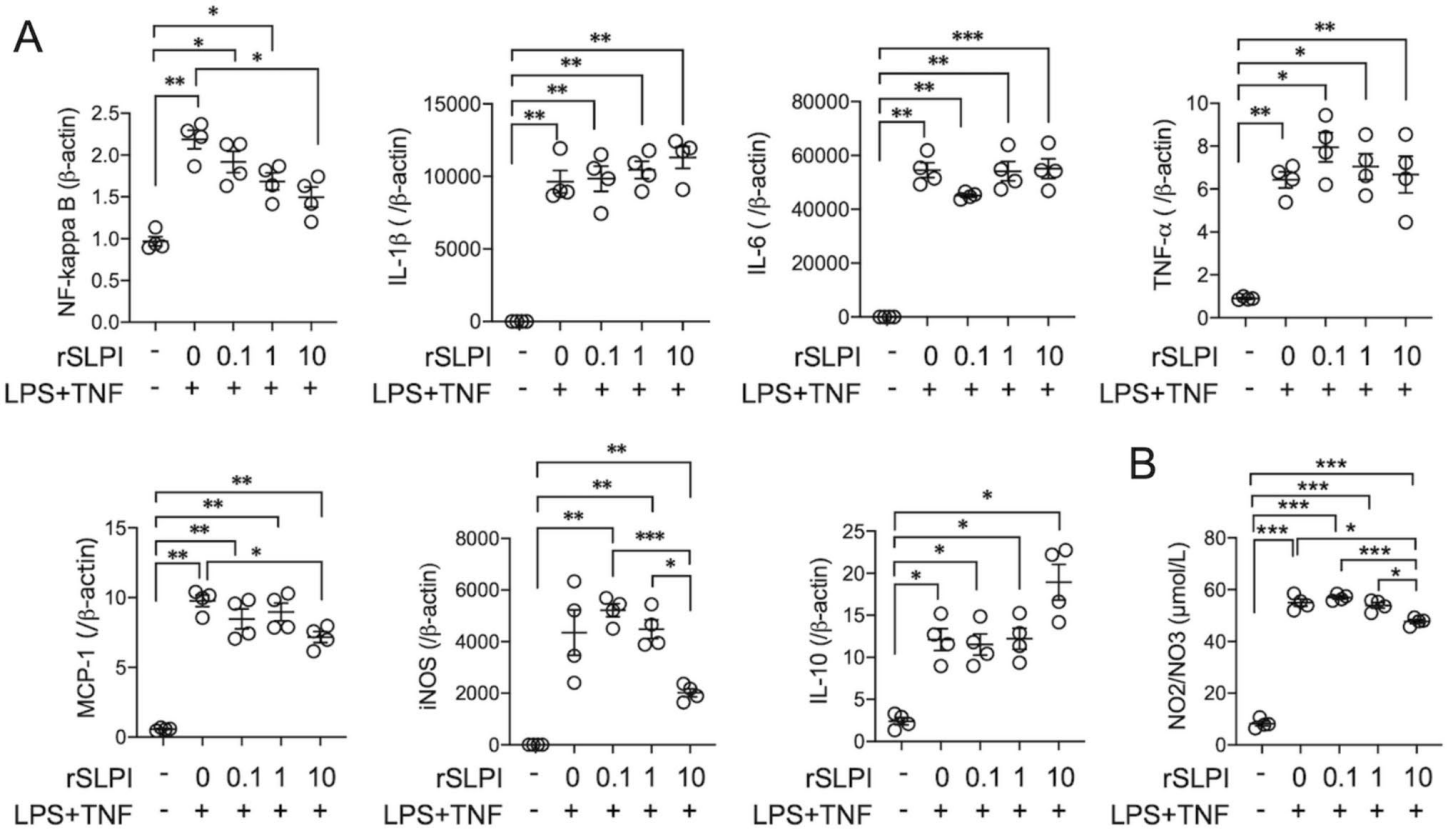
Quantitative analysis of mRNA expression and NO synthesis in inflammatory macrophages stimulated with SLPI. **A** Measurement of the mRNA expression of NF-kB, IL-1β, IL-6, TNF-α, MCP-1, iNOS, and IL-10 (n = 4 each). **B** Measurement of NO synthesis (n = 4). Data are means ± SEM. *p < 0.05; **p < 0.01; ***p < 0.001 assessed by Dunnett’s T3 multiple comparisons test

As shown in Fig. 6A, we investigated changes in mRNA expression levels in macrophages pretreated with different doses of rSLPI. Inflammatory macrophages stimulated with LPS or TNF-α upregulate the mRNA expression of NF-κB, IL-1β, IL-6, TNF-α, MCP-1, iNOS, and IL-10. After pretreatment with rSLPI for 24 h, macrophages exhibited no statistical differences in the mRNA expression of IL-1β, IL-6, or TNF-α, whereas the mRNA expression of NF-κB, MCP-1, and iNOS was downregulated by pretreatment with 10 μg/mL rSLPI relative to that in untreated cells (Fig. 6A). In addition, the volume of NO2/NO3 produced by macrophages decreased in a dose-dependent manner following rSLPI pretreatment (Fig. 6B).

## Discussion

MSC-based therapy has emerged as a promising strategy in the field of regenerative medicine for various diseases, such as graft-versus-host disease, inflammatory diseases, stroke, and cardiovascular diseases [5, 13, 14]. Many reports have shown that MSCs produce pro- and anti-inflammatory cytokines and chemokines and regulate tissue injury responses in a transitory and paracrine manner to orchestrate tissue repair processes [15], whereas MSC therapy has been reported as the most common adverse event, including thromboembolism and fibrosis, in clinical trials [16]. In contrast, MSC-based cell-free therapy has advantages over MSCs therapy because it can avoid these adverse events [17]. However, the molecular mechanisms underlying MSC-based cell-free therapies are not yet well understood. Therefore, it is crucial to investigate secretory factors released by MSCs.

In this study, we profiled MSC-secreted proteins and verified the effectiveness of TAAA treatment using the known anti-inflammatory factors PGRN and SLPI, which are considered to be responsible for the attenuation of TAAA growth by MSC therapy. The abundance of PGRN or SLPI secreted by MSCs was 3.4 ng or 7.1 pg per million MSCs, respectively. The relevance of a dose of 10 mg/kg equates to 10 billion MSCs for PGRN or 4 trillion MSCs for SLPI. The large amounts of rPGRN and rSLPI needed might be related to the contents of the MSC supernatant and the half-life of secretory factors. MSCs secrete not only PGRN and SLPI but also many growth factors and cytokines, and these secretory factors could collectively contribute to the inhibition of aortic aneurysm progression. In addition, engrafted MSCs survive for up to four weeks after administration and can continue to produce secretory factors [7]. Recombinant PGRN and SLPI proteins are expected to have a short half-life after intraperitoneal administration, and their local effects at the aortic aneurysm site are expected to be small. If they can be delivered continuously to the aortic aneurysm site, it may be possible to obtain the effect at a smaller dose.

PGRN, which is a growth factor also referred to as granulin epithelin precursor, plays a critical role in inflammation and wound repair, and also suppresses inflammation by binding to the TNF-α receptor and interrupting TNF-α signaling [18]. In several studies, administration of recombinant PGRN ameliorated renal injury, inflammatory arthritis, myocardial infarction, and acute lung injury [18–22]. The results of the present study showed that rPGRN was less effective than rSLPI at attenuating TAAA growth in mice. Importantly, while PGRN has anti-inflammatory properties, granulins cleaved from PGRN by MMP-9 and elastase have proinflammatory effects [23]. Progressive TAAA is characterized by abundant MMP-9 and neutrophil elastase in the TAAA wall, including the adventitia and thrombus [24, 25]. In this study, rPGRN was administered in the setting of existing TAAA, in which abundant MMP-9 and neutrophil elastase were present. Therefore, it is possible that the administered rPGRN undergoes enzymatic degradation by MMP-9 and neutrophil elastase. However, rPGRN treatment did not increase the expression of inflammatory mediators. Notably, while macrophages in atherosclerotic lesions also express PGRN, those in the atheroma secrete SLPI and inhibit PGRN degradation [26, 27]. PGRN may play an anti-inflammatory role in macrophage infiltration during atherosclerosis, which may suppress the inflammatory process in atherosclerotic plaques. These reports help explain why treatment with rPGRN in our study did not increase the expression of inflammatory mediators or induce the progression of TAAA.

Serine proteinase inhibitors such as SLPI exert their anti-inflammatory effects by inhibiting neutrophil elastase and other leucocyte-derived serine proteinases [28]. In our in vivo experiments, intraperitoneal administration of rSLPI was the most effective in inhibiting TAAA expansion, followed by the combination of rPGRN and rSLPI. This may be due to the fact that conversion of proinflammatory granulins from progranulin is inhibited by SLPI [26], so in mice that were administered both rPGRN and rSLPI, PGRN conversion into granulins may have been moderated by the presence of SLPI. However, the combined administration of rPGRN and rSLPI resulted in a weaker inhibition of effects in inhibiting TAAA growth than rSLPI alone. Zhiheng He et al. suggested that the pathways involved in the expression patterns of PGRN differ between two conditions: (1) when SLPI dominates, PGRN is stable; and (2) when elastase and other proteases are more dominant than SLPI, PGRN is digested into smaller fragments and promotes inflammatory chemotaxis. [29]. PGRN forms a complex with SLPI to inhibit the inflammatory response; however, its binding ratio remains unknown. As mentioned above, MMP-9 and elastase are considered to be dominant in already-formed TAAA. The mild effect of the combination of PGRN and SLPI might be related to the fact that neither the combination of PGRN and SLPI nor PGRN and SLPI alone affected MMP-2 and MMP-9 expression in TAAA tissue homogenates. In addition, considerable differences were observed in the abundance of PGRN and SLPI secreted by MSC, suggesting that a higher PGRN dose may be required for the combination of PGRN and SLPI. Alternatively, the dose of rSLPI administered in this study might have been insufficient to prevent PGRN fragmentation. In contrast, rSLPI administration decreased both the invasion of inflammatory cells and the expression of inflammatory cytokines and chemokines, as well as increased the expression of anti-inflammatory cytokines and maintained the elastic lamellae. These results were supported by the finding that LPS-stimulated macrophages treated with rSLPI exhibited reduced NO production and downregulated expression of several mRNAs, including NF-kB, MCP-1, and iNOS. TAAA is characterized by strong activation of the general inflammatory transcription factor NF-kB [30]. SLPI can enter monocytes and inhibit p65 binding to the NF-kB DNA-binding site [31]. In addition, LPS has been shown to induce iNOS gene expression by initiating the activation of NF-KB [32]. Although the inflammatory role of SLPI in macrophages has been demonstrated, its contribution to other inflammatory cells that contribute to aortic aneurysms, such as T cells and neutrophils, is unknown.

Our study has several limitations. First, the mice received ATII infusion for the first 4 weeks of the experiment, but not from weeks 4 to 8. Our previous study, which used the same experimental design, demonstrated that already formed aortic aneurysms could be temporarily shrunk by MSCs therapy. Therefore, we used the same research design as that used in a previous study to investigate the effects of the MSC-producing factors. However, the expansion and progression of TAAA are delayed in the absence of ATII infusion. To draw firm conclusions about the effect of rSLPI injection on aneurysm progression, intervention with treatments should be considered during the 2–4-week mark, when TAAA progresses dramatically, or when the placement of a second pump to ensure consistent angiotensin II concentrations throughout the experimental timeline is needed. Thus, the results obtained using this experimental protocol are preliminary. Second, angiotensin II-induced AA is a widely used mouse model that exhibits key features of human AA, characterized by the upregulation of inflammation and extracellular matrix remodeling [33]. However, this model is different from human AA in the following aspects: strong implication of macrophage recruitment in the initiation of aneurysm, presence of intramural hematomas, and typical aortic dissection. Although administration of rSLPI inhibits elastin degradation and inflammatory activity, this study must consider the limitations of the mouse model. Additionally, this study did not investigate how SLPI deficiency contributes to the anti-inflammatory effects of TAAA. To prove this, it is necessary to establish genetically modified mice that are double deficient in SLPI or apolipoprotein E. This double-deficient mouse model provides further details and brings us closer to the perspective of clinical applications. Third, all the experiments involved a single injection of one or two recombinant proteins at a concentration of 10 mg/kg after TAAA formation. This concentration was based on several previous studies in which rPGRN was injected intraperitoneally into diseased mice and rSLPI was administered at the same dose [18, 21, 34]. A single injection of rSLPI was effective within the limitations of the study protocol, which did not include ATII infusion for 4–8 weeks. Several studies have reported that the half-life of rSLPI was 4–5 h in rats in aerosol therapy and 1.8 h in the plasma of sheep by intravenous injection [35, 36]. The study did not investigate the volume of rSLPI in the plasma. The relative levels of PGRN and SLPI in mouse circulation are unclear, and it is unclear whether aneurysmal pathogenesis alters the levels of these two proteins in the aorta of mice and humans. The administration doses, injection frequencies, and administration routes for these agents have not been optimized; therefore, further research is necessary.

In conclusion, we identified anti-inflammatory proteins, including PGRN and SLPI, in MSC supernatants and demonstrated that rSLPI administration inhibits TAAA progression in a mouse model using a restricted experimental design. These promising preliminary data present a new approach for the treatment of less-invasive TAAA.

## Supplementary Information

Below is the link to the electronic supplementary material.

Supplementary file1 (DOCX 3728 KB)

## Data Availability

The data underlying this article will be shared on reasonable request to the corresponding author.
