# Supplementary material for: Administration of a recombinant secretory leukocyte protease inhibitor prevents aortic aneurysm growth in mice
Source: Mol Cell Biochem. 2025 Aug 29;481(1):187–97. doi: 10.1007/s11010-025-05374-0 (PMC12906512; doi:10.1007/s11010-025-05374-0)
Supplement: Supplementary file 1 — Supplementary file1 (DOCX 3728 KB) [file 11010_2025_5374_MOESM1_ESM.docx]

**Administration of a Recombinant Secretory Leukocyte Protease Inhibitor Prevents Aortic Aneurysm Growth in Mice**

Aika Yamawaki-Ogata, Masato Mutsuga, Yuji Narita*

Department of Cardiac Surgery, Nagoya University Graduate School of Medicine, Nagoya, Japan

*Corresponding author. Department of Cardiac Surgery, Nagoya University Graduate School of Medicine, 65 Tsurumai-cho, Showa-ku, Nagoya, Aichi 466-8550, Japan. Tel: +81-52-744-2376; fax: +81-52-744-2383; e-mail: ynarita@med.nagoya-u.ac.jp

**Supplementary Figures**

**
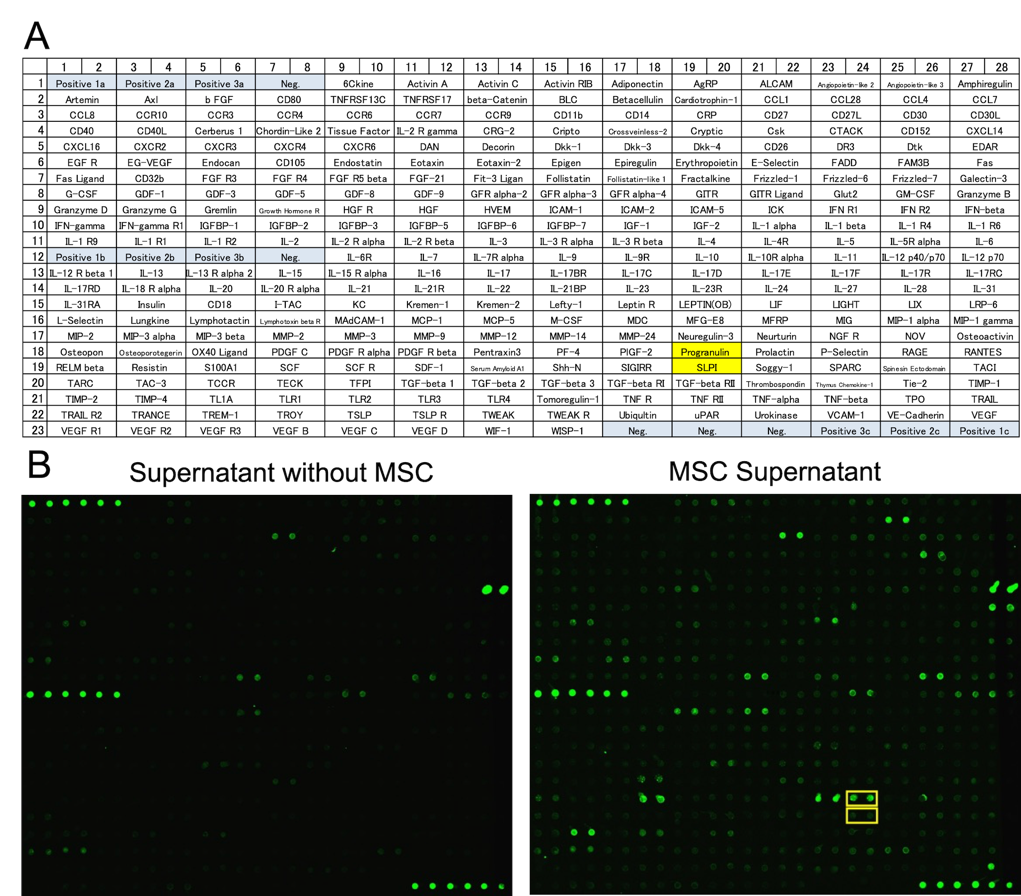
**

**Supplementary Fig. 1** Protein microarray analysis of supernatants with or without MSCs. (A) The map of mouse antibody array L-308. (B) Scanned images of fluorescence signals.


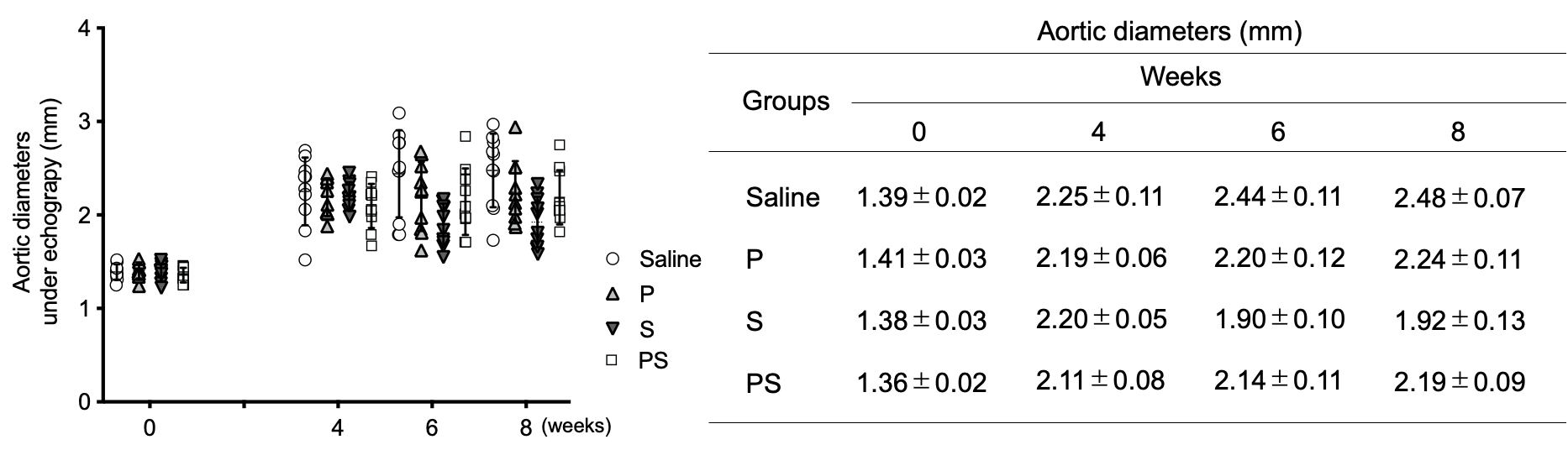


**Supplementary Fig. 2** Individual data of aortic diameters measured by echography (n=10 mice per group). Data were presented as standard error of the mean. Statistical analysis of these data is shown in Fig. 2.

| **Supplementary Table 1.** Primers and PCR characteristics | | | |
| --- | --- | --- | --- |
| Gene | Accession number | Sequence of forward primer and reverse primer |  |
| β-actin | NM_007393 | 5'-AGAGGGAAATCGTGCGTGAC -3' |  |
|  |  | 5'-CAATAGTGATGACCTGGCCGT -3' |  |
| IL-1β | NM_008361 | 5'-CAGGCAGGCAGTATCACTCA-3' |  |
|  |  | 5'-AGCTCATATGGGTCCGACAG-3' |  |
| IL-6 | NM_031168 | 5'-AGTTGCCTTCTTGGGACTGA-3' |  |
|  |  | 5'-TCCACGATTTCCCAGAGAAC-3' |  |
| IL-10 | NM_010548 | 5'-CCAGTTTTACCTGGTAGAAG-3' |  |
|  |  | 5'-TGTCTAGGTCCTGGAGTCCA-3' |  |
| iNOS | NM_010927 | 5'-CCCTTCCGAAGTTTCTGGCAGCAGC-3' |  |
|  |  | 5'-GGCTGTCAGAGCCTCGTGGCTTTGG-3' |  |
| MCP-1 | NM_011333 | 5'-CCACTCACCTGCTGCTGCTA-3' |  |
|  |  | 5'-TGGTGATCCTCTTGTAGCTC-3' |  |
| NF-κB | NM_008689 | 5'-CTGACCTGAGGCCTTCTGGA-3' |  |
|  |  | 5'-GCAGGCTATTGCTCATCACA-3' |  |
| TNF-α | NM_013693 | 5'-TATGGCTCAGGGTCCAACTC-3' |  |
|  |  | 5'-CTCCCTTTGCAGAACTCAGG-3' |  |
